# Role and Mechanisms of Tumor-Associated Macrophages in Hematological Malignancies

**DOI:** 10.3389/fonc.2022.933666

**Published:** 2022-07-07

**Authors:** Yutong Xie, Huan Yang, Chao Yang, Liren He, Xi Zhang, Li Peng, Hongbin Zhu, Lei Gao

**Affiliations:** Medical Center of Hematology, State Key Laboratory of Trauma, Burn and Combined Injury, Xinqiao Hospital, Army Medical University, Chongqing, China

**Keywords:** macrophage, lymphoma, myeloma, leukemia, prognosis

## Abstract

Mounting evidence has revealed that many nontumor cells in the tumor microenvironment, such as fibroblasts, endothelial cells, mesenchymal stem cells, and leukocytes, are strongly involved in tumor progression. In hematological malignancies, tumor-associated macrophages (TAMs) are considered to be an important component that promotes tumor growth and can be polarized into different phenotypes with protumor or antitumor roles. This Review emphasizes research related to the role and mechanisms of TAMs in hematological malignancies. TAMs lead to poor prognosis by influencing tumor progression at the molecular level, including nurturing cancer stem cells and laying the foundation for metastasis. Although detailed molecular mechanisms have not been clarified, TAMs may be a new therapeutic target in hematological disease treatment.

## Introduction

Macrophages are important cellular components of the innate immune system that originate from bone marrow (BM) precursors. Plasticity and diversity are traits of the monocyte-macrophage differentiation pathway. The level of macrophage activation in different locations and at different times indicates the polarization of macrophages. Macrophages are usually polarized into the M1 or M2 type, and these types have different functional characteristics and different abilities to induce T helper cell (Th1 or Th2) responses (1, 2). M1 macrophages are found in settings dominated by Toll-like receptor (TLR) and interferon signaling. M2 macrophages arise in immunity *via* Th2 responses. Both of these macrophage types can indicate the current inflammation and trauma repair statuses.

**Figure 1:**
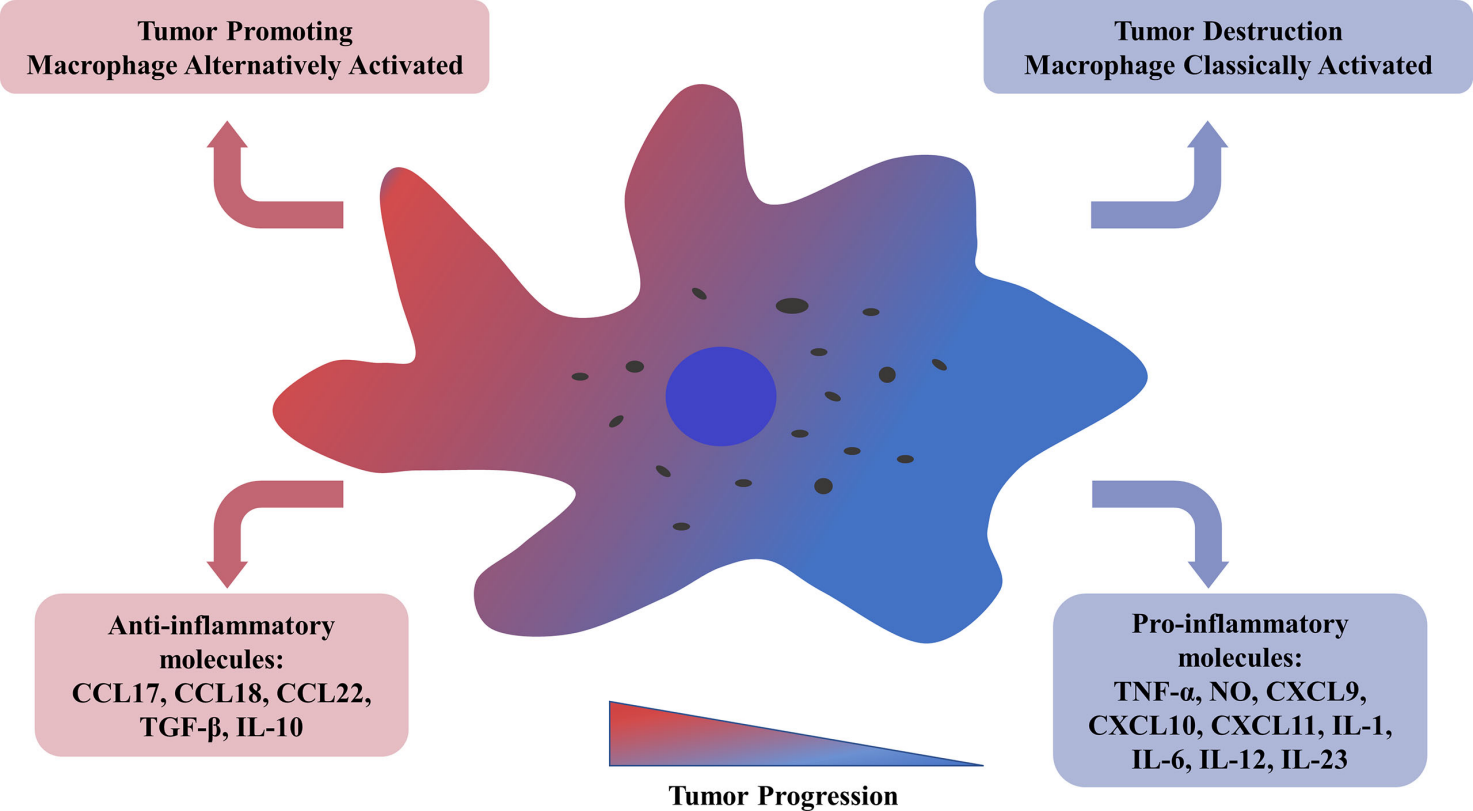
Tumor-associated macrophages could be alternatively activated related to tumor progression and metastasis. M2 TAMs, with a low antigen-presenting capability, get involved in angiogenesis, tumor cell invasion, resistance to therapy, and release of anti-inflammatory molecules, such as CCL17/18/22, TGF-β, IL-10. M1 TAMs could provoke a Th-1 response and secrete pro-inflammatory molecules, such as TNF-α, NO, CXCL9/10/11, IL-1/6/12/23. TNF, tumor necrosis factor; NO, nitric oxide; CXCL, chemokine ligands with CX3-C motif; IL, interleukin; TGF, tumor growth factor.

Recent studies have shown that a group of cells derived from bone marrow called tumor-associated macrophages (TAMs) is recruited to tumors and enhance tumor hypoxia and aerobic glycolysis in solid tumors (3). In particular, some tumor-derived molecules, such as CSF-1 and IL-10, stimulate a considerable proportion of TAMs to differentiate into M2 macrophages (4, 5). Several studies have shown that most kinds of cancer linked to TAMs have poor progression and prognosis (6). M1 type, triggered by GM-CSF, IFN-γ, and LPS, could release pro-inflammatory molecules, such as TNF-α, NO, CXCL9, CXCL10, CXCL11, IL-1, IL-6, IL-12, IL-23; Conversely, M2 phenotype can be activated by M-CSF, TGF-β, IL-4, IL-10, IL-13, which leads to the high secretion of ant nflammatory molecules, such as CCL17, CCL18, CCL22, TGF-β, IL-10 (**Figure 1**) (7–10). In hematological malignancies, like myeloma, lymphoma, leukemia, and other malignancies, macrophages invade tissues and acquire an activated phenotype to participate in disease processes. The relationship between TAMs and Hodgkin lymphoma (HL) has been studied relatively more than the relationships between TAMs and other hematological malignancies. A new study revealed that the adverse overall survival impact of TAMs in classical Hodgkin lymphoma (cHL) is dependent on checkpoint expression, especially on programmed death ligand 1 (PD-L1) and indoleamine 2,3-dioxygenase (IDO-1) expression (11). However, there are few reports on the relationship between TAMs and leukemia. In leukemia, TAMs are referred to as leukemia-associated macrophages (LAMs); they are referred to as acute leukemia-associated macrophages (AAMs) in acute myeloid leukemia (AML) and nurse-like cells (NLCs) in chronic myeloid leukemia (CML). The objective of our review is to discuss the role of macrophages and their activated phenotype in different hematological malignancies.

## Macrophages in Lymphoma

Macrophages can infiltrate malignant tumor tissues. Due to the similarity between lymphomas and solid tumors, many publications have clarified the existence of macrophages in malignant lymphoma. Here, we summarize the mechanisms by which TAMs are involved in angiogenesis, immunosuppression, and activation of tumor cells of HL, T-cell lymphoma and B-cell lymphoma and the clinical prognostic implications.

### Macrophages in Hodgkin Lymphoma

cHL affects young people and is characterized by good prognosis in most cases. There are a small number of neoplastic Hodgkin and Reed-Sternberg (HRS) cells in the microenvironment of cHL with an abundant inflammatory infiltrate of immune cells.

TAMs are linked to adverse prognostic outcomes in HL in a checkpoint-dependent manner. In 1973, Coppleson LW confirmed the existence of macrophages in HL; however, poor prognosis with respect to TAMs was found later by Steidl C et al. (12, 13). The researchers demonstrated that TAM density could predict the treatment outcome by experimenting with paraffin-embedded particles. They also indicated that M2 TAMs could lead to tumor progression and immune escape. Roemer, M. G et al. confirmed that in cHL, the prognostic impact of TAMs on overall survival is checkpoint-dependent. Affected by the genetic/genomic variation of chromosome 9p24.1, PD-1 interacts with PD-L1 and PD-L2 on TAMs (14). IDO-1, which is a tryptophan-catabolizing enzyme, is also expressed by macrophages. Researchers confirmed that large amounts of PD-L1+ and IDO-1+ TAMs lead to adverse survival in patients and that biomarkers of the tumor microenvironment are checkpoint-dependent (11). As Carey et al. reported PD-1^+^ CD4 T cell and CD8^+^ T cell, together with PD-L1+ macrophages and HRS cells played an important role in cHL microenvironment (15). Tislelizumab, a humanized immunoglobulin G4 antiprogrammed cell death protein 1 antibody, binding to Fcg receptor on macrophages, demonstrated a favorable safety outcome for patients with relapsed/refractory cHL in a 3-year follow-up phase II study (16). Werner, L. et al. also confirmed in 2020 that moderate quantities of macrophages were associated with a better prognosis than very low or very high numbers using MYC-positive macrophage detection (17).

Due to the severity of HL progression, accurate prognostic models and clinically relevant biomarkers have become increasingly important. Whiteside TL et al. showed that TAMs were significantly related to primary treatment failure *via* gene expression analysis. They also demonstrated that relapse after auto-HSCT (P=0.008) and reduced progression-free survival (P=0.03) were correlated with CD68^+^ macrophages in HL (18). Subsequently, an increasing number of studies have confirmed the relationship between macrophages and HL. Although CD68 and CD163 are recognized to be specific surface molecules of TAMs, some research has found that the prognostic significance of CD68 is not sufficiently related to clinical outcomes in cHL (19). A phase II clinical trial of CS1001 (PD-L1 inhibitor) of five relapsed or refractory (R/R) cHL elucidated that multiplex immunofluorescence staining was less intense for CD163 than CD68 (20).However, scholars from Korea found that CD163 is a better prognostic marker of macrophages in cHL (21). New reports suggested that HL patients with the highest M2 TAM count using CD163 as an M2 polarization marker had reduced disease-free survival and overall survival (11). These findings indicate that CD163 is better than CD68 as a prognostic marker for TAMs in HL.

The abovementioned findings indicate that it is likely that the effect of TAMs on outcome in HL may be related to potential changes in macrophage polarization. HRS cells can differentiated TAMs towards M2 phenotype by secreting molecules such as TGF-β and IL-13 (22). Ruella et al. cultured M2-like phenotype macrophages from monocytes, together with HDLM-2 cells and GM-CSF. The results showed that these M2 macrophages, expressed CD163, CD206, PD-L1, inhibited the growth of human CD19 chimeric antigen receptor (CAR) T cells, which suggests an unsatisfactory therapeutic effect of CAR T cell. CD123 expresses on macrophages in the microenvironment of HL, suggesting that CD123-targeted therapies might impact on the tumor microenvironment (23).It was previously shown that TAMs can activate HL cell proliferation through the STAT3 pathway (24). The STAT3 pathway also induces macrophage polarization toward the M2 phenotype (25). The STAT3 pathway can be activated by macrophage-derived factors such as epidermal growth factor (EGF), IL-6, and IL-10 (26–29). In cHL patients, IL-10 is also regarded as a marker of tumor burden and as an unfavorable host-tumor factor (30, 31). IL-10 was reported to promote the poor overall survival of cHL *via* genetic regulation of the tumor microenvironment. Single-nucleotide polymorphisms (SNPs) in IL-10 can be regarded as prognostic markers in adult cHL. Furthermore, the percentage of macrophage activating factor (MAF)-expressing cells can change, which suggests a role of these cells in determining the host genetic background that induces macrophage polarization and an indirect role in microenvironment shaping (32). The PI3K-Akt pathway is also involved in HL pathogenesis (33). PI3K inhibitor RP6530 decreases the release of lactic acid by downregulates the metabolic regulator pyruvate kinase muscle isozyme 2 (PKM2) (34). A first-in-human phase I, open-label study of Tenalisib (RP6530) enrolled 35 patients across 11 dose levels with R/R hematological malignancies correlated well with clinical outcome and further phase I/II studies are being undertaken to evaluate the efficacy (35). Later, Locatelli et al. found that downregulating lactic acid released by HRS favors M2 type macrophage. They also found that the blockade of PI3K could lead M1-type macrophages to transition into the M2 type, which suggests a new therapeutic strategy to treat patients with HL (36). However, how these mechanisms truly affect macrophage polarization remains unclear.

### Macrophages in Non-Hodgkin Lymphoma

#### Macrophages in Diffuse Large B-Cell Lymphoma

Diffuse large B-cell lymphoma (DLBCL) accounts for 30-40% of non-HL clinical cases (5). TAMs play an active role in the progression of DLBCL. Some studies have confirmed that TAMs and specifically M2-TAMs are linked to poor prognosis in DLBCL and central nervous system DLBCL (37, 38). In *in vitro* assessment of the progression of DLBCL, M2 TAMs were found to affect the overexpression of legumain by disrupting the extracellular matrix and promoting angiogenesis (39).

In DLBCL, the relationship between CD68^+^ TAMs and overall survival varies. Some studies have shown that there is no relationship between TAMs and prognosis, whereas others have reported a significant influence (40, 41). Many studies have shown that CD163+ TAMs and the CD163/CD68 ratio are linked to clinical outcome (37, 42, 43). Specifically, it is thought that different therapeutic options could exert different influences on TAMs. 85% to 95% of *de novo* DLBCL patients express PD-L1, correlated with macrophages and STAT3 expression (44). Pollari et al. collected tumor tissue from 74 primary testicular lymphoma patients, and examined macrophage markers, T-cell markers, B-cell marker, and checkpoint molecules, illustrating that PD-1- PD-L1 signaling have a promising role in clinical outcome (45).

In DLBCL, TAMs have been demonstrated to produce cytokines such as C5a, IL-6 and TNF-α to activate the Stat3 and NF-kB pathways (46). A new study investigated the relationship between neuron-specific enolase (NSE) levels and the prognosis of DLBCL. The researchers found that the protein expression of, which mediates nuclear p50 translocation with subsequent dysfunction of classical nuclear factor-κB (NF-κB), thereby promoting M2 polarization and shifting the role of macrophages, was increased in DLBCL (47). This mechanism is related to the IRF/STAT signaling pathway of macrophage polarization. Polarization can be skewed toward the M1 phenotype *via* STAT1 signaling and toward the M2 phenotype *via* STAT6 signaling. Two adaptors, MyD88 and TRIF, regulate signaling downstream of TLR4, which ultimately activates NF-κB, a pivotal transcription factor influencing M1 macrophage activation. M1 macrophage polarization is predominated by the NF-κB and STAT1 pathways, which play a proinflammatory role (48, 49). It is well known that the most common therapy for DLBCL is CHOP (cyclophosphamide, doxorubicin, vincristine, and prednisone) or the combination of rituximab and CHOP (R-CHOP) chemotherapy (50, 51). R-CHOP combined with granulocyte-macrophage colony-stimulating factor (GM-CSF) prolongs the survival of elderly DLBCL patients (52, 53). Zhang et al. first reported the antitumor, macrophage polarization-related molecular mechanisms by which GM-CSF affects CHOP and R-CHOP therapy in 2021 (54). The researchers found that GM-CSF induced repolarization of M2 macrophages to increase M1 macrophages, providing ideas for how macrophages mediate the AKt pathway, a well-characterized pathway in DLBCL.

#### Macrophages in Follicular Lymphoma

Follicular lymphoma is a common indolent B-cell lymphoma characterized by a slow clinical course that is usually considered incurable. Research on macrophages in follicular lymphoma has mostly focused on predicting overall survival. Kridel R et al. found that two patient groups treated with different therapies showed an opposite correlation between M2 TAM density and prognosis (55).Some studies have suggested that the number of CD68+ TAMs is related to the prognosis of follicular lymphoma (56). Another study found that patients with an increased number of CD68+ TAMs had longer survival (57). Although the number of CD163+ macrophages can predict the prognosis of patients with follicular lymphoma, their impact depends on the treatment that the patients received from a study involved 395 samples treated with rituximab, cyclophosphamide, doxorubicin, vincristine, and prednisone, and randomized to rituximab maintenance or observation (55). Furthermore, a recent meta-analysis revealed that high CD68+ LAM numbers, diffuse patterns of FOXP3+ regulatory T (Treg) cells and PD1+ cells, and high PD-L1 cell numbers are adverse factors leading to early transformation of follicular lymphoma. A study on the immune microenvironment of follicular cell lymphoma showed that immune infiltrate diversity portends good clinical efficacy in follicular lymphoma, implying that a rich immune microenvironment in follicular lymphoma is important (58).

Colony-stimulating factor-1 (CSF-1) and its receptor CSF-1R have been thoroughly studied in follicular lymphoma, and the results have indicated that macrophages can be a new therapeutic target because CSF-1 is one of the most important recruitment factors for macrophage polarization. Regarding solid tumors, glioma has been reported to be cured by treatment targeting the CSF-1R/CSF-1 axis. A recent study demonstrated that CSF-1R inhibition by PLX3397 has a higher impact on M2 macrophages than on M1 macrophages and leads to their repolarization toward an M1-like phenotype (59).

#### Macrophages in Marginal Zone B-Cell Lymphoma

Splenic marginal zone lymphoma (SMZL) is also an incurable indolent small B-cell lymphoma usually occurring in elderly people (average age of 65 years old) (60). Escape from immune control is the main change leading to exacerbation of the disease, characterized by abundant T-cells in the periphery but low numbers in the stroma, which surrounds large amounts of tumor cells (61). Chen and Mellman et al. suggested that SMZL is characterized by an inflamed phenotype, with the presence of intratumoral infiltration of T-cells into the tumor microenvironment in addition to myeloid-derived suppressor cells (MDSCs) and tissue-associated macrophages, which is closely related to poor overall survival (61). SMZL with an inflamed phenotype features the expression of PD-L1 as a mechanisms for immune escape; PD-L1 is colocalized with CD163, a marker of alternatively activated macrophages (62).

#### Macrophages in Peripheral T-Cell Lymphoma

Peripheral T-cell lymphoma (PTCL) is an aggressive form of lymphoma in Asia that usually leads to poor overall survival (63). Early research found that in acute T-cell leukemia/lymphoma, CD68^+^ TAM infiltration exists; however, this feature is not related to poor prognosis and angiogenesis. In contrast, the number of CD163^+^ TAMs was found to be associated with for the prognosis of T-cell lymphoma (64, 65). Iqbal J et al. analyzed the influence of CD68 expression on the promotion of macrophage differentiation by GATA-binding protein 3 (GATA3). GATA3 and T-box family transcription factor (T-bet) are Th1 and Th2 cell differentiation markers, respectively, and T-bet-positive PTCL has a better prognosis than GATA3-positive PTCL (66, 67).

Cutaneous T-cell lymphoma (CTCL) is a heterogeneous group of T-cell lymphomas located in the skin in which macrophages behave as M2 macrophages (68, 69). In a study of CTCLs, a high number of CD163^+^ M2 TAMs was linked to a poor clinical prognosis and was correlated with the level of soluble IL-2 (65). IL-10 is reported to be higher than average in CTCLs. IL-10 has been confirmed to increase the expression of PD-L1 to induce anti-inflammatory regulation. Xuesong W et al. proved that IL-10 is not only a biomarker but also a key cytokine in macrophage polarization that leads to tumor growth and can inhibit effective cutaneous T-cell lymphoma therapy (70). A retrospective study of 205 patients published in 2020 showed that an increased level of IL-10 is an independent factor that indicates poor overall survival, a low complete response rate and a higher early relapse rate (71).

## Macrophages in Myeloma

Multiple myeloma (MM) is a B-cell hematological tumor characterized by a large number of malignant plasma cells in the bone marrow. MM cells are highly dependent on the bone marrow microenvironment and can create an immunosuppressive microenvironment conducive to tumor growth by secreting cytokines or directly contacting surrounding immune cells. Macrophages are abundant in the bone marrow of patients with MM and can promote the growth, proliferation and drug resistance of tumor cells and participate in the formation of an immunosuppressive microenvironment.

TAMs can negatively influence MM growth and progression, leading to a poor outcome. Angiogenesis is a major feature of MM and features stimulation of angiogenic factors by plasma cell, inducing the transformation of monoclonal gammopathy of undermined significance (MGUS) into MM (72). TAMs can secrete proangiogenic cytokines like VEGF and TNF-α and express proangiogenic enzymes such as cyclooxygenase-2 (COX-2) (73, 74). There has been some research progress related to microRNAs involved in the bone marrow microenvironment. Exosome-derived miR-let-7c promotes angiogenesis by polarizing M2 macrophages in the MM microenvironment (70). Macrophages also regulate tumor growth by controlling cell metabolism. One hypothesis is that M2 macrophages can inhibit PGK1 phosphorylation by secreting IL-6 to disrupt the connection between macrophages and tumor cells (75).

Elevated microvessel density has been linked to CD163-positive TAMs and CD68/CD163 double-positive M2 TAMs. Andersen et al. suggested that in MM patients, CD163 expression was higher in bone marrow than in blood samples, and high CD163 expression correlated with a poor prognosis and a higher International Staging System (ISS) stage. Moreover, an increased number of CD163^+^ TAMs has also been found to be a powerful predictor of poor prognosis in MM in the era of novel drugs. CD163 and inducible NO synthase (iNOS) expression have been combined with ISS stage as new prognostic factors. Furthermore, increased expression of CD206, a soluble M2 macrophage marker, indicates reduced overall survival (76–79). Sanyal et al. found a novel cell surface marker for M2 macrophages MS4A4A which includes CD20 (MS4A1), FcRβ (MS4A2) and Htm4 (MS4A3), suggesting immunotherapeutic potential in the treatment of MM (80).

CCL2 is a critical molecule that recruits monocytes and induces inflammation (81). The inactivation of CCL2-CCR2 was found to reduce tumor growth in solid neoplasms. The chemokine CCL2 was found to promote macrophage infiltration in the MM bone marrow microenvironment and to encourage proliferation (82). De Beule and colleagues revealed that AZD1480, a Janus kinase 2 (JAK2) inhibitor, was correlated with protumor effects *via* the STAT3 pathway in 5T33MM cells (78, 83). A recent study found that increased CCL2 induces MCPIP1 expression *via* the JAK2-STAT3 signaling pathway, which promotes tumor growth (79, 84). Trabectedin, a drug that kills monocytes and macrophages, triggers antiangiogenic activity by suppressing CCL2 and VEGF production. Due to this effect, the potential of trabectedin as a targeted agent in anti-MM therapeutic strategies has been proposed (85). CSF1R blockade significantly inhibits myeloma-associated macrophage polarization to the M2 type, implying that CSF1R-blocking antibodies could be a new tool for MM therapy (86).

## Macrophages in Leukemia

Since lymphoma is similar to solid tumors, research in lymphoma is relatively common. However, leukemia is unique. There are significant pathological differences between leukemia and solid tumors, and thus, studies of macrophage properties and actions in leukemia are lacking. Compared to solid tumors, hematological malignancies have a unique immunological microenvironment. Leukemia originates from leukemic stem cells (LSCs), and these LSCs maintain the hematopoietic microenvironment and hematopoietic stem cell (HSC) survival and function, which supports LSC proliferation through complex signals. Leukemia is propagated by LSCs, which cannot be totally eradicated and persist, ultimately leading to recurrence (87). TAMs, existing in the microenvironment of different types of leukemia are called LAMs.

### Macrophages in Acute Lymphocytic Leukemia

Acute T-cell leukemia is characterized by infection with human T-cell leukemia virus. Komohara et al. showed that CD163^+^ M2 macrophages are closely associated with the progression of T-cell acute lymphocytic leukemia (T-ALL) (88). Recently, researchers reported that an inhibitor of the M-CSF receptor may suppress the stimulation of macrophages, which can be used as a therapeutic strategy (89). A JAK2/FLT3 inhibitor, pacritinib, was found to block CSF-1R to improve the microenvironment (89). Further research found that CSF-1R signaling paves the way for TAM recruitment and obstructs TAM proliferation in a T-ALL mouse model (90). The CXCR4/CXCL12 axis was found to inhibit TAM polarization toward the M1 phenotype. Some preclinical studies have demonstrated that the CXCR4 inhibitor plerixafor improves the clinical scores of T-ALL (91).

In B-cell acute lymphocytic leukemia (B-ALL), there are few studies about macrophages. MDSCs and Treg cells have become the focus of B-ALL research in recent years given their mutual relationship. MDSCs have emerged as a great contributor to tumor angiogenesis, drug resistance, and the promotion of tumor metastases (92). Recent studies have shown that MDSCs can continue to differentiate into TAMs in the tumor environment and can be divided into an M1 subgroup, which inhibits tumor growth, and an M2 subgroup, which promotes tumor growth. MDSCs consist of two types: monocytic MDSCs (MO-MDSCs) and polymorphonuclear MDSCs (PMN-MDSCs) (93). A recent study found that PMN-MDSCs and Treg cells play important roles in maintaining the immune-suppressive state of B-ALL, which means that they may be independent predictors of B-ALL progression. However, the relationship between peripheral Treg cells and MDSCs has not been fully recognized (94). Lineage reprogramming could be a promising future treatment in B-ALL therapy, and such a strategy was shown to eliminate the leukemogenicity of Ph-positive B-ALL cells and turn them into macrophage-like cells *in vitro* (91).

### Macrophages in Acute Myeloid Leukemia

A German scholar named the macrophages in AML as AML-associated macrophages (AAMs). In AML, which factors induce macrophage polarization remains unclear. Al-Matary YS et al. proved that LAMs exert an important influence on the overall survival and drug resistance of AML patients. Their results suggest that leukemic cells and the microenvironment can induce the proliferation and infiltration of monocytes and macrophages and promote their differentiation into AAMs. The main reason for relapse in AML is LSCs, which can be supported by AAMs *via* extracellular matrix remodeling, angiogenesis, and lymphangiogenesis (95). AAMs highly express Gfi1, which polarizes M1 phenotype macrophages into M2 macrophages to suppress the immune system (96). In addition, a growth factor-independent transcriptional repressor was found to reprogram LAMs toward the antitumor state. The leukemia hematopoietic microenvironment is complex and includes fibroblasts, macrophages and other components. Variations in the hematopoietic microenvironment in leukemia have not been reported. There is mounting evidence that illustrates that the AML microenvironment can re-educate monocytes and macrophages to transition into the M2 phenotype. Mussai et al. provided the first reports showing that arginase II secreted from primary AML blasts reeducates healthy donor-derived monocytes toward an M2-like phenotype, as demonstrated by upregulation of CD206 (97).

The AML microenvironment has tissue-specific heterogeneity. In the MLL-AF9 AML mouse model, splenic LAMs more often exist in the M2 phenotype, while bone marrow LAMs more often exist in the M1 phenotype. AML creates an immunosuppressive microenvironment. By demonstrating an arginase-dependent ability of AML, Mussai et al. polarized surrounding monocytes into a suppressive M2 type macrophage. The researchers also found that repolarization of LAMs through targeting of the SAPK/JNK pathway and IRF7-SAPK/JNK pathway by interferon regulatory factor 7 (IRF7) can prolong the survival of AML mice, providing regarded a new immunotherapy strategy against leukemia (96, 98). Keech et al. showed a high degree of leukemia burden in MLL-AF9 AML mice and that nonmalignant and AML bone marrow macrophages display a decrease in M1 macrophage markers (99). It has also been found that monocytic leukemia zinc-finger (MOZ) is a direct target of miR-223 promoting monocyte-to-macrophage development and M1 polarization (100). A recent study showed that peritoneal resident macrophages in mice with AML induced by MLL-AF9 show an M2-like phenotype (101). These results strongly suggest that the leukemia microenvironment may enhance the immunotherapy effect in AML by affecting the apoptosis and killing ability of macrophages. Switching M1 to M2 is through lasting exposure to polarizing molecules or direct cell-to-cell contact between macrophages and cancer cells (102). Smirnova, T. further proved that in the presence of GM-CSF, inhibiting CSF1R could repolarization macrophage, thus improving the efficiency of AML therapy, which indicated a promising therapeutic method to modulate macrophage phenotype (103).

### Macrophages in Chronic Lymphocytic Leukemia

Chronic lymphocytic leukemia (CLL) is characterized by the accumulation of CD5^+^ B cells in blood, secondary lymphoid organs and bone marrow. Burger JA et al. found that nurse-like cells (NLCs) derived from blood could protect CLL B-cells from apoptosis through stromal cell-derived factor-1 (104). In fact, NLCs are CLL-specific TAMs characterized by expression of the markers CD68 and CD163 (105). It is unknown why NLCs increase the survival rate and drug-induced apoptosis of CLL cells. Boissard et al. reported that LFA-3 appeared to have an adverse influence on prognosis in an exploratory cohort of 60 CLL patients receiving frontline immunochemotherapy (105). IFN-γ was found to reprogram NLCs into the M1 state (106). The JAK2/FLT3 inhibitor pacritinib restrains the CSF-1R signaling pathway, thus preventing the generation and survival of NLCs (107). Edwards V DK et al. showed that significant synergy was observed when combining CSF1R inhibitors with two current CLL therapies that block the signaling pathway of the tumor cell-intrinsic B-cell receptor (108). The proinflammatory switch of NLCs plays an important role in modulating the CLL microenvironment. Trabectedin also induced an antileukemia effect in a CLL mouse model by depleting TAMs *via* the CCL2-CCR2 signaling axis (109). The CSF1 receptor also participates in antineoplastic activation by interfering with leukemic cell and NLC interactions (107).

### Macrophages in Chronic Myeloid Leukemia

Previous studies have shown that M2-type macrophages are the predominant infiltrate in the bone marrow microenvironment of CML patients, with their functions being dominant, and the number of positive cells increases gradually with the progression of the disease (110). By CSF1/CSF-1/M-CSF pathway, autophagy can be induced through the differentiation from human monocytes to macrophages. Researchers found that P2RY6 agonist activated CSF-1 treated monocytes differentiation to promote autophagy induction in some CMML patients (111). Researchers found high accumulation of CD68^+^, CD163^+^ and CD206^+^ macrophages in bone marrow biopsy samples. Macrophages have been reported to increase the cytotoxicity of natural killer (NK) cells against solid tumor cells. In CML bone marrow aspirates, there are higher proportions of macrophages and NK cells. Choo et al. found that mycoplasma-infected CML cells were protected from NK cytotoxicity by macrophages, which was related to macrophage-mediated maintenance of NK cells (112). Besides, the polarization of the M2-like macrophages was found to be associated with K562‐derived exosomes in CML (113). This can be a new sight into leukemia-derived exosomes in the development of leukemic niches.

## TAM-Targeted Therapeutic Strategies

**Figure 2:**
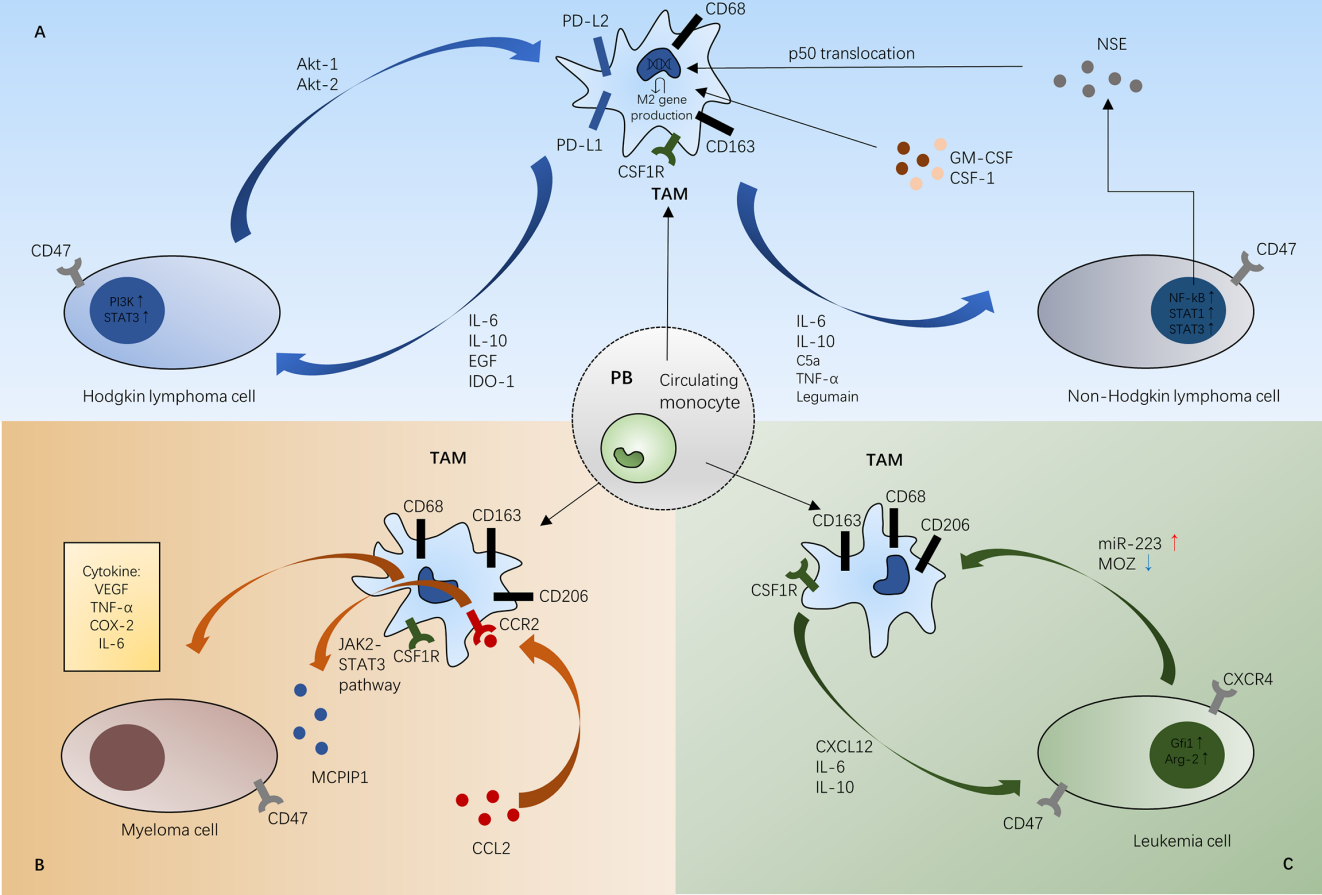
Schematic representations of mechanisms between TAMs and tumor cells in Hodgkin lymphoma and non-Hodgkin lymphoma **(A)**, myeloma **(B)**, and leukemia **(C)**. In the different tumor microenvironment, TAMs infiltrate different tumor tissue to promote tumor growth. **(A)** In Hodgkin lymphoma, TAMs can activate HL cell proliferation through the STAT3 pathway and PI3K-Akt pathway. Besides, M1 macrophage polarization can be predominated by the NF-κB and STAT1 pathways in non-Hodgkin lymphoma. **(B)** CCL2 induces MCPIP1 expression *via* the JAK2-STAT3 signaling pathway in the MM bone marrow microenvironment. TAMs can also secrete proangiogenic cytokines like VEGF in MM microenvironment. **(C)** In the leukemia microenvironment, CSF-1R signaling paves the way for TAM recruitment. Gfi1 polarizes M1 phenotype macrophages into M2 macrophages to suppress the immune system and MOZ is a direct target of miR-223 promoting monocyte-to-macrophage development and M1 polarization. STAT3, signal transducer and activator of transcription; EGF, epidermal growth factor; IDO-1, indoleamine 2,3-dioxygenase; CSF1R, colony-stimulating factor receptor; CSF-1, colony-stimulating factor-1; GM-CSF, granulocyte macrophage-colony stimulating factor; TNF- α, tumor necrosis factor-α; NSE, neuron-specific enolase; VEGF, vascular endothelial growth factor; COX-2, cyclooxygenase-2; MOZ, monocytic leukemia zinc-finger; Gfi1, growth factor independent 1; Arg-2, arginase-1; PB, peripheral blood; TAM, tumor-associated macrophage.

TAMs may be a therapeutic target because they are involved in cancer progression and characterized by unique transcriptional profiles (**Figure 2**) (114). It has been reported that M1-like TAMs have an antitumor effect, while M2-like TAMs have a protumor effect (115). Therefore, induction of TAM polarization from the M2 to M1 phenotype could be a therapeutic strategy to treat hematological malignancies. The different signaling pathways in TAM polarization mainly include five pathways: the JAK/STAT signaling pathway, Notch signaling pathway, PI3K/Akt signaling pathway, TLR/NF-κB signaling pathway and hypoxia-dependent signaling pathway. In addition, some natural compounds also downregulate M2 polarization (116). Our summary of the pathways shifting macrophage polarization is shown in **Table 1**.

**Table 1:** Pathways shifting macrophage polarization in hematological malignancies.

Disease	Mechanism of Action	Results
HL	PI3K-Akt pathway	Leading M1 type macrophage to M2 type
DLBCL	GM-CSF synergistic enhancement effect	Enhancing M1 polarization from M2
DLBL	NSE protein mediates nuclear p50 translocation *via* IRF/STAT signaling pathway	Promoting M2 polarization and migration ability of macrophage
PTCL	GATA3-dependent mechanism	M2 macrophage differentiation
FL	CSF-1R inhibition by PLX3397	repolarization towards an M1-like phenotype
MM	Inactivation of CCL2-CCR2	Macrophage bone marrow homing, proliferation, and polarization
MM	STAT3 pathway	A Janus kinase (JAK)2 inhibitor was correlated to the pro-tumor effect
MM	Exosome-derived miR-let-7c promotes angiogenesis	Polarizing M2 macrophages in MM microenvironment
MM	CSF1R blockade	Inhibits myeloma-associated macrophage polarizing to M2 type
T-ALL	CXCR4/CXCL12 axis	Inhibiting TAM polarization towards M1 phenotype
AML	Demonstrating an arginase-dependent ability of AML	Suppressive M2-like phenotype *in vitro*
AML	Expressing Gfi1	Polarizing M1 phenotype macrophage into M2
AML	MOZ Forms an Autoregulatory Feedback Loop with miR-223	Promoting monocyte-to-macrophage development and M1 polarization
AML	Inhibiting CSF1R, in the presence of GM-CSF	Reprogramed MΦ orientation and promoted myeloblast apoptosis
CLL	IFN-γ	Reprogramming tool to polarize NLCs to M1 state
CLL	CSF-1R signaling inhibition	LAMs polarization blocking

TAM-targeted therapy towards the conjugation of antibodies and ligands to the therapeutic molecule carrier. The role and prognostic markers of TAMs, which can be also recognized as crucial receptors in hematological macrophages, is shown in **Table 2**. CD163 receptor on the surface of macrophage can recognize the complex of hemoglobin (Hb) and plasma haptoglobin (Hp). Conjugating the anti-cancer drug dichloroacetic acid (DCA) to the Hb-Hp complex targets the delivery of DCA into cancerous monocytes and scavenges cancer cells (117, 118). Wang et al. suggested that CD71 is a invariably marker and highly expressed in different subtypes of leukemia cells based on which they designed a biomimetic carrier for precision delivery of As^III^, As@Fn nanomedicine, to bind to HL-60 AML leukemia cells characterized by CD71 (119). This finding gives us a new perspective into ferritin-based targeted therapy connected to hemoglobin targeted therapy delivered to TAMs. More anti-tumor drugs and clinical research are expected to extend our therapeutic scope.

**Table 2:** The role and prognostic markers of TAMs in hematological malignancies.

Disease	Reference	Marker	Number of Patients	Survival Correlation
cHL	Karihtala et al. (11)	PD-L1, IDO-1	130	High proportions of PD-L1 and IDO-1 TAMs are associated with unfavorable outcomes
	Carey et al. (15)	PD-L1	180	Increased PD-L1 expression had superior PFS
	Kayal et al. (19)	CD68	100	CD68 TAM marker does not have prognostic value
	Suh et al. (21)	CD68, CD163	144	CD163 is a better prognostic marker of macrophages than CD68
DLBCL	Marchesi et al. (37)	CD68/CD163	61	High CD68/CD163 M2 TAM is correlated to unfavorable prognostic factors
	Wang et al. (42)	CD163	355	LMR was negatively correlated with CD163 M2 TAM
	Cencini et al. (43)	CD68/CD163	37	CD68+/CD163+ TAM have a prognostic role for IPI ≥ 2 DLBCL patients receiving R-CHOP
	McCord et al. (44)	PD-L1	777	PD-L1 did not identify high-risk in *de novo* DLBCL
	Pollari et al. (45)	PD-L1/CD68	74	High PD-L1/CD68 macrophages predict favorable survival
FL	Kridel et al. (55)	CD163	186	CD163 TAM predict outcome dependent on treatment received
	Kelley et al. (56)	CD68	94	CD68 TAMs is related to the prognosis
	Bjoürck et al. (57)	CD68	57	Patients with an increased number of CD68 TAMs had longer survival
	Kridel et al. (55)	CD163	395	CD163 macrophages can predict the prognosis depending on the treatment
SMZL	Vincent-Fabert et al. (62)	PD-L1	54	Exhibiting inflammation with the expression of PD-L1
PTCL	Sugaya et al. (65)	CD163	28	CD163 M2 TAMs was linked to a poor clinical prognosis
	Iqbal et al. (66)	CD68	372	CD68 TAM differentiation by GATA3 related to worse prognosis
ATLL	Saito et al. (64)	CD204	58	CD204 TAMs were closely associated with lymphoma cell proliferation
MM	Andersen et al. (76)	CD163	131	Soluble CD163 was found to be a prognostic marker
	Suyanı et al. (77)	CD163	68	High MVD was found to be associated with increased CD163 TAM
	Chen et al. (78)	iNOS, CD163	240	iNOS and CD163 TAMs as independent prognostic factors
	Wang et al. (79)	CD163	198	High CD163 TAM correlate with poor prognosis

PFS, progression-free survival; LMR, lymphocyte-to-monocyte ratio; MVD, microvessel density.

The relationship between overexpression of CD47, a glycoprotein highly expressed in myeloid and lymphoid malignancies, and poor prognosis is under investigated (120). CD47 induces immune escape by binding to the receptor SIRPα to inhibit macrophage phagocytosis and improve T-cell cytotoxicity (121). Advani et al. confirmed that the Hu5F9-G4 antibody has a synergistic effect with rituximab in 22R/R DLBCL and follicular lymphoma patients, indicating that blocking the CD47 immune checkpoint, a so-called “don’t eat me” signal, could exert antitumor effects (122). A recent study demonstrated that the addition of rituximab to CHOP chemotherapy improved the overall outcome of DLBCL patients (123). *In vitro* experiments illustrated that novel fully human anti-CD47 monoclonal antibodies increased macrophage-mediated phagocytosis and improved the prognosis of AML models (124). CC-90002 is an anti-CD47 antibody to block CD47-SIRPα interaction and enhance macrophage-mediated killing ability. However, a phase 1 study of anti-CD47 monoclonal antibody CC-90002 in patients with R/R AML and high-risk MDS still suggested insufficient evidence in clinical activity as expected in spite of well preclinical effect (125).

Trabectedin together with Zoledronic acid are two drugs used to kill tumor cell and TAMs. Trabectedin targets DNA transcription, leading to DNA double strand breaks and cell cycle blockade, which demonstrates a potent anti-tumor effect against Hodgkin Reed Sternberg cells. Tumors of trabectedin-treated mice had fewer TAMs, reducing secretion of CCL5, M-CSF, IL-6, IL-13 in HRS cells (126). Zoledronic acid is a potential therapy to change tumor microenvironment, affecting the secretion of CCL5 and IL-6. In prostate cancer, zoledronic acid repolarizes M2 macrophages to M1 type, exhibiting antitumor effect (127).

Lenalidomide has been proven to influence the tumor microenvironment by improving T-cell and NK-cell function (128). In mouse MM models, lenalidomide was proven to promote M2 macrophage depletion and affected the Th1/Th2 balance (129). The effect of adding ASCT to triplet therapy (lenalidomide, bortezomib, and dexamethasone [RVD]) in patients with multiple myeloma was associated with longer progression-free survival than RVD alone in a phase 3 clinical trial (NCT01208662) (130). Martino et al. reported a retrospective multicenter analysis of 600 RRMM patients treated with the combination of lenalidomide and dexamethasone (KRd) with a 79.9% overall response rate after a median of 11 KRd cycles (131). Pyridoxine, a specific treatment in AML, induces monocyte-macrophage death and apoptosis in THP-1 cells to play an antitumor role (132).

TAK-981 is the first-in-class small-molecular inhibitor of SUMOylation in clinical trials. Small ubiquitin-like modifier (SUMO) is a ubiquitin-like protein superfamily, promoting inflammatory responses and expressing IFN-1.By blocking SUMOylation, TAK-981 allowing NK cell activation and M1 polarization to enhance antibody-dependent cellular cytotoxicity (ADCC) and antibody-dependent cellular phagocytosis (ADCP) *via* upregulating IFN-1. Nakamura Et al. showed a preclinical research that TAK-981 and rituximab in xenograft models of human B cell lymphoma have antitumor effect (133). Assouline et al. further proved in a phase 1b/2, open-label, dose-escalation and expansion study that TAK-981 plus rituximab resulted in promising clinical activity (ORR 29%) in the R/R NHL (134). Combination of TAK-981 with anti-CD38 antibody daratumumab also resulted in protective clinical antitumor immune response (135). TAK-981 increased phagocytic activity of macrophages and natural killer cell cytotoxicity *via* IFN-1 signaling, which could be a promising treatment for patients with hematological malignancies (136).

## Conclusion

Macrophages have attracted wide attention in solid tumor research, and their role in hematologic malignancies should also remain a focus. TAMs are referred to as LAMs, AAMs or NLCs in hematologic malignancies. Distinct microenvironments induce different molecular mechanisms of TAMs. The microenvironments of hematologic malignancy can induce the activation of macrophages into type M2 macrophages, which play an important role in angiogenesis, immunosuppression, and the activation of tumor cells. Strategies to reprogram the polarization of macrophages are new therapeutic options in hematologic malignancies.

## Author Contributions

The manuscript was conceptualized by LG and XZ. YX wrote the majority of the manuscript and HY cowrote the manuscript. The figures were drawn by CY and LP. HZ and LH produced the table. All authors contributed to the article and approved the submitted version.

## Funding

This work was supported by the Chinese National Natural Science Foundation (Grant No. 82170161), Chongqing National Natural Science Key Foundation (Grant No. cstc2019jcyj-zdxmX0023), the Clinical Medicine Innovation Project of Army Medical University (Grant No. 2018JSLC0034) and Medical Frontier Project of Xinqiao Hospital (Grant No. 2018YQLY007).

## Conflict of Interest

The authors declare that the research was conducted in the absence of any commercial or financial relationships that could be construed as a potential conflict of interest.

## Publisher’s Note

All claims expressed in this article are solely those of the authors and do not necessarily represent those of their affiliated organizations, or those of the publisher, the editors and the reviewers. Any product that may be evaluated in this article, or claim that may be made by its manufacturer, is not guaranteed or endorsed by the publisher.
